# The complete chloroplast genome sequence of potato wild relative species, *Solanum nigrum*

**DOI:** 10.1080/23802359.2016.1250133

**Published:** 2016-11-11

**Authors:** Tae-Ho Park

**Affiliations:** a Department of Horticulture, Daegu University, Gyeongsan, South Korea;; b Institute of Life and Environment, Daegu University, Gyeongsan, South Korea

**Keywords:** Chloroplast, genome, genome sequence, *Solanum nigrum*

## Abstract

*Solanum nigrum* is a wild non-tuber-bearing species belonging to Solanaceae family. The complete chloroplast genome of *S. nigrum* was constituted by *de novo* assembly using a small amount of whole genome sequencing data. The chloroplast genome of *S. nigrum* was 155,432 bp in length and consisted of 25,289 bp of a pair of inverted repeats, 18,402 bp of small single copy, and 85,852 bp of large single copy regions. A total of 139 genes were annotated including 96 protein-coding genes, 39 tRNA genes, and four rRNA genes. Maximum likelihood phylogenetic analysis with 25 Solanaceae species revealed that *S. nigrum* is grouped with *S. melongena*.

*Solanum nigrum*, a wild hexaploid species, is a relative to potato (*S. tuberosum*) and widely grows in South Korea. It has several desirable characteristics such as various resistances against biotic and abiotic stresses, especially late blight resistance (Colon et al. 1993), and has been used as a resource for potato breeding. However, breeding using the wild species has been limited due to its sexual incompatibility with *S. tuberosum* caused by the different ploidy level of the genome and endosperm balance number (Oritz & Ehlenfeldt 1992; Cho et al. 1997). Therefore, protoplast fusion of the two different species has been attempted to breed new varieties of potato (Binding et al., 1982; Barsby et al., 1984) and somatic hybridization via protoplast fusion provides opportunity to overcome sexual barriers for interspecific gene transfer with late blight resistance in potato breeding program (Eijlander & Stiekema 1994; Zimnoch-Guzowska et al. 2003).

The *S. nigrum* (DU_GS8) was collected in Gyeongsan, South Korea, and has been stored at Daegu University, South Korea. An Illumina paired-end (PE) genomic library was constructed with total genomic DNA according to the PE standard protocol (Illumina, San Diego, CA) and sequenced using an Illumina HiSeq2000 at Macrogen (http://www.macrogen.com/kor/), South Korea. Low-quality bases with raw scores of 20 or less were removed and then ∼1.9 Gbp of high-quality of PE reads were assembled by a CLC genome assembler (ver. 4.06 beta, CLC Inc., Rarhus, Denmark) with the parameters of minimum (300–1000 bp) autonomously controlled overlap size as described by Kim et al. (2015). The principal contigs representing the chloroplast genome were retrieved from the total contigs using Nucmer (Kurtz et al. 2004) with the chloroplast genome sequence of *S. bulbocastanum* (NC007943) as the reference sequence (Daniell et al. 2006). The representative chloroplast contigs were arranged in order based on BLASTZ analysis (Schwartz et al. 2003) with the reference sequence and connected to a single draft sequence by joining the overlapping terminal sequences. The chloroplast genes were predicted using DOGMA (Wyman et al. 2004) and BLAST searches.

The complete chloroplast genome of *S. nigrum* (GenBank accession no. NC028070) was 155,432 bp in length including 25,589 bp inverted repeats (IRa and IRb) regions separated by small single copy (SSC) region of 18,402 bp and large single copy (LSC) region of 85,852 bp with the typical quadripartite structure of most plastids, and the structure and gene features were typically identical to those of higher plants. A total of 139 genes with an average size of 571 bp were annotated including 96 protein-coding genes with an average size of 754 bp, 39 tRNA genes, and four rRNA genes. An overall GC content was 38%.

Phylogenetic analysis was performed using chloroplast coding sequences of *S. nigrum* and 24 published species in Solanaceae family by a maximum likelihood method in MEGA 6.0 (Tamura et al. 2013). According to the phylogenetic tree, *S. nigrum* belonged to the same clade in *Solanum* species. However, interestingly it was close to *S. melongena* and separated with six other *Solanum* species including *S. tuberosum* (Figure 1).

**Figure 1.**
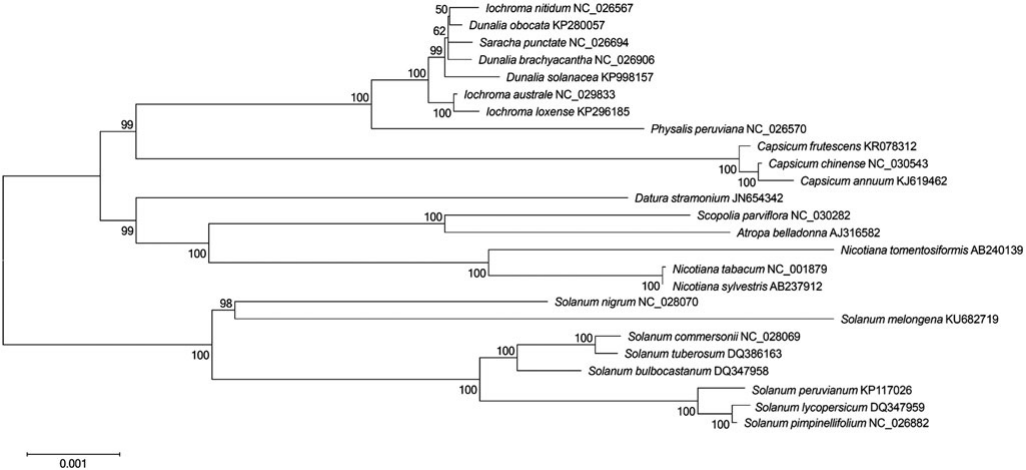
Maximum likelihood phylogenetic tree of *S. nigrum* with 24 species belonging to the Solanaceae based on chloroplast protein coding sequences. Numbers in the nodes are the bootstrap values from 1000 replicates.
